# Mating system shifts and transposable element evolution in the plant genus *Capsella*

**DOI:** 10.1186/1471-2164-15-602

**Published:** 2014-07-16

**Authors:** J Arvid Ågren, Wei Wang, Daniel Koenig, Barbara Neuffer, Detlef Weigel, Stephen I Wright

**Affiliations:** Department of Ecology and Evolutionary Biology, University of Toronto, Toronto, ON M5S 3B2 Canada; Department of Molecular Biology, Max Planck Institute for Developmental Biology, Tübingen, 72076 Germany; Department of Botany, University of Osnabrück, Osnabrück, 49076 Germany

**Keywords:** Transposable elements, Mating system, Brassicaceae, *Capsella*, Reference genome bias

## Abstract

**Background:**

Despite having predominately deleterious fitness effects, transposable elements (TEs) are major constituents of eukaryote genomes in general and of plant genomes in particular. Although the proportion of the genome made up of TEs varies at least four-fold across plants, the relative importance of the evolutionary forces shaping variation in TE abundance and distributions across taxa remains unclear. Under several theoretical models, mating system plays an important role in governing the evolutionary dynamics of TEs. Here, we use the recently sequenced *Capsella rubella* reference genome and short-read whole genome sequencing of multiple individuals to quantify abundance, genome distributions, and population frequencies of TEs in three recently diverged species of differing mating system, two self-compatible species (*C. rubella* and *C. orientalis*) and their self-incompatible outcrossing relative, *C. grandiflora*.

**Results:**

We detect different dynamics of TE evolution in our two self-compatible species; *C. rubella* shows a small increase in transposon copy number, while *C. orientalis* shows a substantial decrease relative to *C. grandiflora*. The direction of this change in copy number is genome wide and consistent across transposon classes. For insertions near genes, however, we detect the highest abundances in *C. grandiflora*. Finally, we also find differences in the population frequency distributions across the three species.

**Conclusion:**

Overall, our results suggest that the evolution of selfing may have different effects on TE evolution on a short and on a long timescale. Moreover, cross-species comparisons of transposon abundance are sensitive to reference genome bias, and efforts to control for this bias are key when making comparisons across species.

**Electronic supplementary material:**

The online version of this article (doi:10.1186/1471-2164-15-602) contains supplementary material, which is available to authorized users.

## Background

In plants, transposable element (TE) abundance ranges from around 20% in the compact *Arabidopsis thaliana* genome [1] to over 80% in the maize genome [2]. Although it has long been clear that TE content varies enormously across taxa, the extent of, and evolutionary reasons for, TE variation among closely related species is less clear [3, 4]. Where whole genome sequences are available, comparisons have been limited to two species [5–8] and where more species were compared, analyses were typically restricted to one or a few TE families [9–15]. Until recently, the lack of large-scale genomic data for closely related species has precluded comprehensive tests. This problem is rapidly diminishing with the increase in available whole genome sequences, allowing theoretical models to be tested across genomes and across species at a scale not previously possible [7, 16–18].

According to several models, mating system is expected to play an important role in driving the evolutionary dynamics of TEs [19, 20]. There are two main reasons for this. First, the spread of TEs may be inhibited by a lack of outcrossing [21–24]. Second, self-regulation of transposition is more likely to evolve in selfers than in outcrossers and host-silencing mechanisms are more likely to spread to fixation with greater linkage to the active element [25]. Therefore, all else being equal, outcrossing species are predicted to maintain a higher abundance of TEs than selfing species. Alternatively, the expected reduction in the effective population size (N_e_) in selfers relative to outcrossers [26, 27] and the associated reduction in the efficacy of selection may lead to fewer TEs in the genomes of outcrossers [9, 28]. Furthermore, if selection is mainly due to chromosomal rearrangements caused by between-element (ectopic) recombination, lower heterozygosity in selfers may lead to relaxed selection and as a consequence TE accumulation [9, 24, 29]. Empirical evidence to date, while limited, provides some support in favour of TE loss following the evolution of selfing [19]. In particular, the *Arabidopsis thaliana* genome has consistently fewer insertions in comparison with its outcrossing congener *A. lyrata* [6, 30, 31], which may partly be driven by more efficient host silencing via small RNAs [32]. Moreover, there is some evidence suggesting smaller genome sizes in selfers compared with related outcrossers [19, 33] (but see [34]). However, whether the evolution of selfing will generally result in an increase or decrease in TE copy number, and the timescale over which TE abundance evolves, remains unclear.

**Figure 1:**
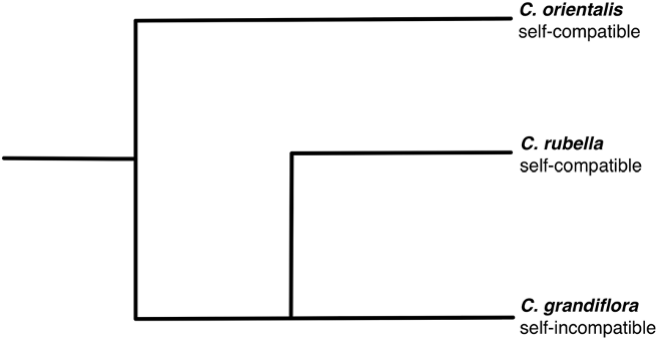
**Phylogenetic relationships within the** ***Capsella*** **genus.** For an comprehensive review of the evolutionary history of the genus, see [38].

The plant genus *Capsella* provides a promising system to study interspecific variation in TE abundance and distribution. *Capsella*, which diverged from *Arabidopsis* somewhere between 6 and 20 million years ago [35–37], is a relatively small genus within the mustard family (Brassicaceae). Furthermore, the members of the genus vary in mating system ([38]; Figure 1). The sequencing of the genome of the self-compatible *Capsella rubella* and comparisons with its self-incompatible closest relative *C. grandiflora* suggested that *C. rubella* has experienced a global reduction in the efficacy of natural selection on non-synonymous polymorphisms, but without evidence for major shifts in transposable element abundance during the less than 200,000 years since divergence [39].

Here, we expand the mating system comparisons to population samples from three characterized *Capsella* species, two self-compatible species and one self-incompatible outcrosser. Self-compatible species have evolved at least twice through the divergence from a self-incompatible outcrossing ancestor similar to *C. grandilflora* (2n = 2x = 16), resulting in the recent *C. rubella* (2n = 2x = 16; divergence time 50–200, 000 years [40, 41]) and *C. orientalis* (2n = 2x = 16; divergence time unknown, but believed to be older than *C. rubella* [38]). *C. grandiflora* is geographically restricted to a glacial refugium in northern Greece and has a stable effective population size (N_e_ ~ 600,000), with relatively little population structure [42, 43]. *C. rubella* spans the Mediterranean region, while *C. orientalis* stretches from the far eastern parts of Europe, through the South Urals and western Mongolia to northwestern China [38]. The effective selfing rate in *C. rubella* has been estimated to be 0.90–0.97 [42]. Although the selfing rate in *C. orientalis* has not been quantified, very low allozyme variability suggests that the species is predominately selfing [38]. For an extensive review of the evolutionary history of the genus, see [38]. We quantify abundance, population frequencies, and genome wide distributions of TEs across the three species and use the results to examine whether the variation is consistent with the effects of mating system outlined above. We also discuss the residual uncertainty of using the reference genome of one species in a comparative study of TE abundance and distribution, and steps that may be taken to address the issue.

## Results

### Distribution of TE insertions in *Capsella*

We quantified TE abundance using the paired-end read mapping approach of Kofler et al. [44]. Paired-end Illumina reads from multiple individuals from all three species (8 *C. grandiflora*, 10 *C. orientalis*, and 24 *C. rubella* individuals) were mapped to a repeat-masked *C. rubella* reference genome [39], and a TE database [39] with repeats from seven Brassicaceae species (*A. thaliana* (reference accession Col-0 and accessions Ler, Kro-0, Bur-0, and C24 from the 1001 Arabidopsis genomes project), *Arabidopsis lyrata*, *Arabis alpina*, *Brassica rapa*, *Capsella rubella*, *Eutrema halophila*, *Schrenkiella parvulum*). Individual TE insertions were identified by cases where one read maps to a TE and a second to a unique genomic location [44]. The TE database comprised 4,261 different TE sequences. While the Kofler et al. [44] method was originally implemented for pooled population samples, we sequenced individual samples, and used estimates of insertion frequencies to call insertions as heterozygous or homozygous. We performed several tests to assess the suitability of this approach, all of which confirm our general conclusions (see Methods for details).

**Figure 2:**
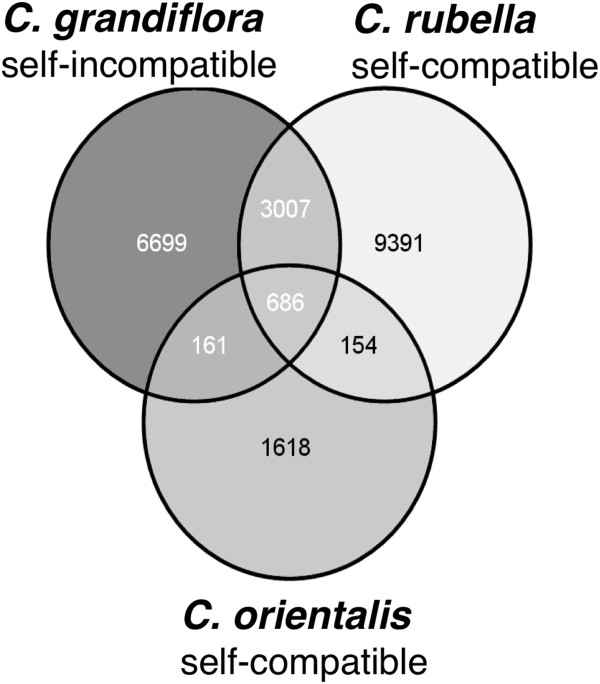
**Venn diagram with the number of unique and shared TE insertion sites in three** ***Capsella*** **species.**

**Figure 3:**
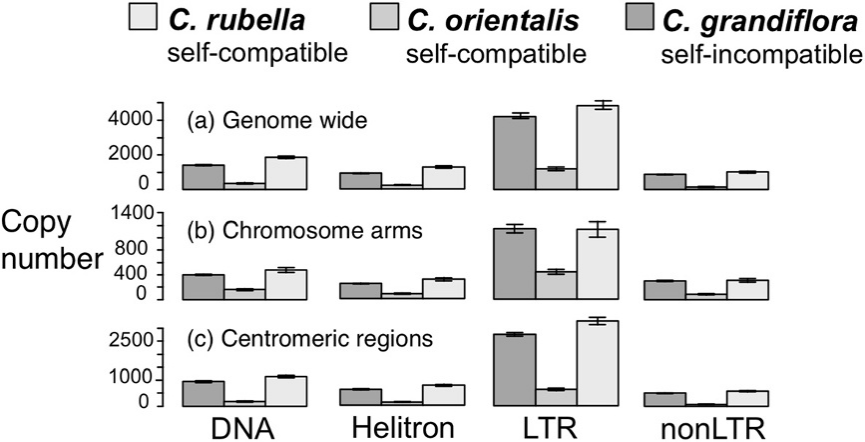
**Average TE copy number in the three** ***Capsella*** **species genome wide (a), on chromosome arms (b), and in centromeric regions (c). Error bars are ± 1 standard error.**

We identified 21, 716 unique insertions across the three species. Of all insertions considered, the majority (approximately 80%) are unique to one species (Figure 2). There is a strong consistency in the distribution of copies among TE families. LTR elements are the most common type, making up roughly 59% of all TEs; DNA elements comprise 19%; Helitron and non-LTR elements approximately 11% each (Figure 3a). The closely related *A. thaliana* and *A. lyrata* also show no difference in the relative abundance of families [31]. In both genera, non-LTRs are the smallest contributors to the TE load in the genomes. However, in *Arabidopsis* the DNA elements dominate (including Helitrons) making up over 55% of all TEs, consistent with the reported expansion of the *Basho* Helitrons in *A. thaliana* [45].

Since using the *C. rubella* assembly as the reference genome may bias our analysis we used several approaches to assess whether our results were robust to the effect of reference genome bias. To begin assessing this issue we compared the proportion of reads from all species that mapped to the non-pericentromeric regions of the main chromosome scaffolds of the *C. rubella* reference genome. Reassuringly, these proportions do not differ dramatically between the three species (*C. rubella*, 30%; *C. grandiflora,* 27%; *C. orientalis,* 25%)*.* We discuss additional approaches to control for reference genome bias in more detail in connection with the relevant results below.

### Abundance of TEs

The estimated mean number of TE insertions varied between the three species (Kruskal-Wallis chi-squared = 62.0227, df = 2, p < 0.00001; Figure 3). Estimated TE copy number is lowest in self-compatible *C. orientalis*, highest in the outcrosser *C. grandiflora* and self-compatible *C. rubella*. This pattern holds true for all four classes of TE (LTRs, non-LTRs, DNA, and Helitrons). The mean copy number is slightly higher in *C. rubella* compared to *C. grandiflora* (Wilcoxon signed rank test with continuity correction, V = 528450463, p = 0.004385). This difference is due to a higher number of DNA elements (V = 17017897, p = 0.004798) and Helitrons (V = 9236731, p < 0.00001). Moreover, within-species variation in TE abundance is highest in *C. rubella* (Additional file 1: Figure S1).

To better characterize between-species differences, we separately examined copy numbers in gene-rich chromosome arms, where we expect selection against TE insertions to be strong, and those in centromeric regions, where selection should be weak [46]. Indeed, all families show significantly higher densities in pericentromeric regions (in *C. orientalis* only LTR and Helitrons) (Mann–Whitney test, p < 0.05). When comparing species, significant lower TE numbers are still apparent on the chromosome arms for *C. orientalis*, but we see no significant difference between *C. rubella* and *C. grandiflora* (Kruskall-Wallis rank sum test, chi-squared = 15.4656 df = 2, p < 0.0001).

A central concern with our results is the bias that might arise from using *C. rubella* as the reference genome in the analysis. Reference genome bias can come in two forms: 1) a greater ability to find insertion sites due to higher mapping of flanking regions, and 2) a greater representation of TEs from the reference species. Although all of our species are closely related, and species differences in the percentage of reads mapping to the genic regions are small, our general patterns of TE abundance follow the phylogenetic pattern expected if reference genome bias is playing a role with species closest to *C. rubella* showing the greatest TE abundance. To further address the first concern we first took advantage of the previously generated Illumina-based *de novo* assemblies of *C. grandiflora* [39] and the close outgroup *Neslia paniculata* [39], and also performed a *de novo* assembly of *C. orientalis* (see Methods for details). To confirm the lower numbers of TEs in *C. orientalis*, we redid the analysis for the *C. orientalis* and *C. grandiflora de novo* assemblies with a TE database including only insertions identified in the *C. orientalis* assembly. The assemblies of the two species should be of similar quality and using a *C. orientalis* biased TE database should reverse the bias that might arise from using our larger TE database, as *C. grandiflora* may be expected to share more insertions with *C. rubella*. Taking this approach, we still detect significantly lower TE numbers in *C. orientalis* (Additional file 2: Figure S2a, generalized linear model with Poisson distribution, z = −20.6, p < 0.00001). A caveat is that the *de novo* assembly of *C. orientalis* is very TE-poor to begin with.

Second, to address the two concerns while removing any reference bias, we mapped all species against the *de novo* assembly of *N. paniculata*, using a TE database based only on insertions identified in *A. thaliana* and *A. lyrata*. Although the number of insertions identified was dramatically reduced, we saw the same pattern of differential TE abundance, in particular fewer TEs in *C. orientalis* (Additional file 2: Figure S2b, generalized linear model with Poisson distribution, z = −9.394, p < 0.00001). Thus, while reference genome bias likely plays some role in the estimated magnitude of the between-species differences in TE abundance, on balance the data generally support the inferred low TE numbers in *C. orientalis*.

### TE insertions near genes

**Figure 4:**
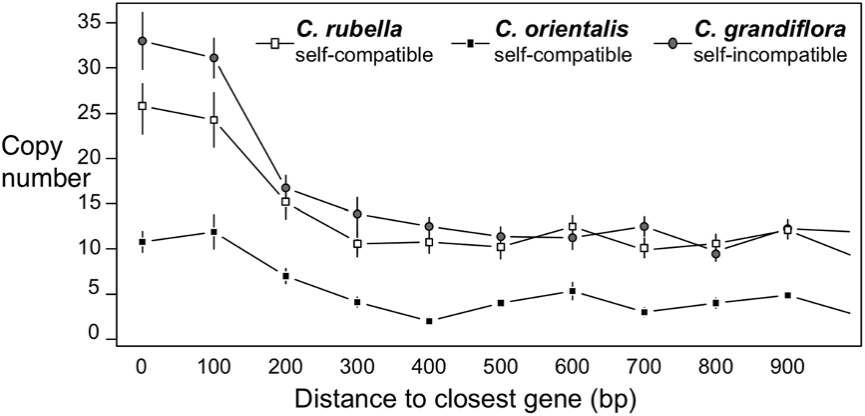
**Average TE copy number in 100 bp bins near their closest gene in the three** ***Capsella*** **species.** Error bars are ± 1 standard error.

Under several population genetic models, TEs are expected to be rapidly removed from gene-rich regions [47]. Comparing TE copy number in regions near genes may therefore provide insights about the number of recent insertions in a given genome. Using the gene annotation from *C. rubella* [39], we calculated the distance to the closest gene from the beginning (or end, whichever was closest) of each insertion. We find a trend to more insertions within 1,000 bp of genes in *C. grandiflora* compared to the other species (Figure 4), with *C. orientalis* again having the lowest TE density near genes. *Capsella orientalis* is significantly different from the other two species (Kruskal-Wallis chi-squared = 1021.348, df = 2, p < 0.00001). *Capsella grandiflora* shows significantly higher abundance than *C. rubella* in regions within 200 bp of the nearest gene (generalized linear model with Poisson distribution, z = −4.789, p < 0.00001).

Focusing the analysis on regions close to genes, where problems with read mapping should be minimized, also allows us to address the second way in which reference genome bias may occur: a greater ability to find insertion sites due to higher mapping of flanking regions. Again, the lower number of TEs near genes in *C. orientalis* is consistent with our general conclusions.

### Frequency distributions of TEs

**Figure 5:**
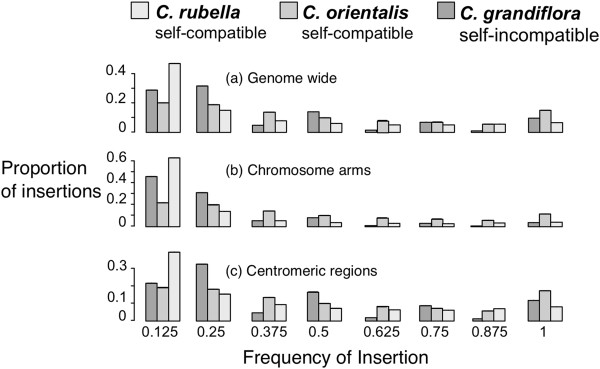
**Histogram of population frequencies of TEs in the three** ***Capsella*** **species genome wide (a), on chromosome arms (b), and in centromeric regions (c).** 95% confidence intervals based on 200 bootstraps are plotted but too small to be seen.

We used the presence or absence of all identified insertions across individuals to calculate population frequency distributions for all three species. The TE distribution of *C. orientalis* is distinct from that in *C. rubella* and *C. grandiflora* (Kruskal-Wallis chi-squared = 399.93, df = 2, p < 0.00001; Figure 5). This is true also when ignoring fixed insertions (Kruskal-Wallis chi-squared = 481.33, df = 2, p < 0.00001). *C. orientalis* has the highest number of fixed insertions and the lowest proportion of rare insertions, consistent with a low or no contemporary accumulation of TEs and a general genome wide reduction in diversity across the genome. Consistent with the hypothesis of relaxed selection near centromeres, both *C. rubella* and *C. grandiflora*, but not *C. orientalis* differ in their frequency distributions between chromosome arms and centromeric regions, with a significant excess of common insertions in the centromeric regions (Wilcoxon Rank sum test, both p < 0.05).

## Discussion

Here, we report results from a whole genome study of TE abundance and distributions in multiple individuals in three species from the plant genus *Capsella*. Comparing population samples from the outcrosser *C. grandiflora* to two of its self-compatible relatives allows us to begin to empirically dissect the population and genome wide effects of a mating system shift in driving TE evolution.

The evolution of selfing does not appear to have had the same effect in the two self-compatible *Capsella* species. Perhaps the most striking result of this study is the consistently lower TE copy numbers in the self-compatible *C. orientalis*. This reduction is apparent for all families and in both centromeric regions and along chromosome arms (Figure 3), as well as for recent insertions near genes (Figure 4). Furthermore, *C. orientalis* shows a detectable absence of rare and an excess of common TE insertions (Figure 5). The present transposition rate thus appears to be very low in *C. orientalis*. TE accumulation is known to be a key driver of genome size evolution in plants [3, 48] and this reduction in transposition rate may in part explain why *C. orientalis* has the smallest genome in the genus [38].

In contrast to *C. orientalis*, *C. rubella* does not appear to be TE poorer than *C. grandiflora*. Instead, there is a trend of an increase in copy number, which seems to be due to higher accumulation in centromeric regions (Figure 3), although this observation may also be due to poorer mapping of the other species in these regions. However, when we consider only insertions near genes, which are where recent insertions tend to reside, *C. grandiflora* has a higher abundance than *C. rubella* (Figure 4). *Capsella rubella* also has the highest excess of rare insertions, although this trend is most pronounced along the chromosome arms (Figure 5). This may reflect an increase in transposition rate or be a product of the recent population bottleneck *C. rubella* experienced in conjunction with the evolution of selfing [40, 49].

What would determine whether selfing leads to a net accumulation or loss of TEs? One important factor is likely to be the age of the selfing lineage [19]. As outlined in the Introduction, selfing will reduce the effective population size and this reduction following the shift to selfing may initially result in an increase in fixation rates compared to the outcrossing relative. However, over time, the lack of outcrossing means that any new (deleterious) insertion that arises in either lineage will have a harder time spreading in the selfing lineage. As a consequence, we may observe different effects on selfing in a young and an old lineage. Here, we detected fewer TEs in *C. orientalis*, but a slight increase in *C. rubella. Capsella orientalis* diverged from *C. grandiflora* before *C. rubella* did, suggesting that it may have been self-fertilizing for longer. It is important to note, however, that the speciation event and the evolution of selfing may not have occurred simultaneously. This, for example, is the case in *A. thaliana* [50–53], where the evolution of selfing apparently occurred a long time after the speciation event. Although the shift to self-fertilization can occur both within a lineage and in conjunction with a speciation event, recent work by Goldberg and Igić indicate that the shift is ten times as likely to be associated with a speciation event than to occur within a lineage [54]. While it is not clear whether the evolution of selfing in *C. orientalis* coincided with the speciation event as it did in *C. rubella* [40], the very recent origin of *C. rubella* and the very low species wide allozyme variability in *C. orientalis* [38] suggest that *C. orientalis* may have been selfing longer than *C. rubella*. Proper dating of the origin selfing in *C. orientalis* should be the focus of future work.

We undertook several approaches to control for reference genome bias in copy number estimation. There is a clear effect of such bias in that the relative copy-number difference estimated depends strongly on which reference genome is being used for mapping. On the one hand, the *C. rubella* genome is by far the highest-quality reference genome, and in most cases we detect the highest copy numbers using this genome as the reference (compare Figure 3 with Additional file 2: Figure S2). However, taking this approach may also maximize the bias, causing an exaggerated assessment of copy number differences between species. Nevertheless, the patterns observed, particularly with our bias-free mapping to the *N. paniculata* genome, do suggest that our general conclusions may be robust to assembly and mapping differences. Ultimately, long-read data integrated with higher-quality assemblies of all *Capsella* species will be important for validating the results reported here.

## Conclusions

Taken together our results suggest that the effects of mating system on transposon evolution may vary from case to case. A candidate factor determining the direction of the effect may the age of the selfing lineage. Finally, cross-species comparisons of transposon abundance are sensitive to reference genome bias and caution must be applied when using re-sequencing approaches.

## Methods

### Sampling and sequencing

Samples from all species come from a large range of their species distributions (Additional file 3: Table S1). *C. grandiflora* samples come from 12 populations, with one individual sampled per population, spanning the native range in Greece. The thirteenth sample was cross between two other populations. For *C. orientalis* we obtained samples from five previously described populations [38], with two individuals per population sampled. After growth in the University of Toronto greenhouse for several months, DNA from leaf tissue from all samples was extracted using a modified CTAB protocol [55]. Sequencing was done at Genome Quebec Innovation Centre using the Illumina Genome Analyzer platform 121 (Illumina, San Diego, California, USA). *Capsella rubella* samples came from across its geographical range and were grown and sequenced at the Max Planck Institute for Developmental Biology, Germany. The median average coverage was 20x for *C. orientalis*, 39x for *C. grandiflora*, and 22x for *C. rubella*. Sequences are available on the Sequence Read Archive (http://www.ncbi.nlm.nih.gov/sra): *C. orientalis* (Accession number SRP041585), *C. grandiflora* (Accession number SRP044121), and *C. rubella* (Accession number PRJEB6689).

### Genome assemblies

For *C. rubella*, we used the recently completed reference genome [39]. We also took advantage of the Illumina-based *de novo* assemblies of *C. grandiflora* and *N. paniculata* prepared for that analysis (for details see [39]). The *C. orientalis* assembly was prepared from 17.6 Gb of 108 bp Illumina paired-end reads in ten libraries. Reads were assembled into contigs using the Ray (v 2.1.0) assembler [56] with a Kmer of 31 under 20 multiple cores (N50 ~ 25 kb). Contigs shorter than 500 bp were discarded after scaffolding. For further assembly details see Additional file 3: Table S2. *De novo* assemblies are available on CoGe (https://genomevolution.org/CoGe/): *C. orientalis* (Genome ID 24033), *C. grandilfora* (Genome ID 24068), and *N. paniculata* (Genome ID 24067).

### Identification of unique TE insertions

To detect TE insertions across the re-sequenced genomes we used PoPoolationTE [47]. The method requires three things: (1) annotated reference genome, (2) a library of TE sequences, and (3) paired-end sequence data. The strength of this approach is that it allows the identification of insertions not present in the reference genome. Here, we used the recently completed *Capsella rubella* genome [39], as well as the Brassicaceae TE database generated as part of the genome annotation (for details of TE annotations in the *C. rubella* genome see [39]). We ran the pipeline using default settings on 108 bp paired-end Illumina samples from 8 *C. grandiflora*, 10 *C. orientalis*, and 24 *C. rubella* individuals.

PoPoolation typically requires DNA from pooled samples from multiple individuals; it then uses the read mapping results to estimate population wide frequencies. Here, instead of using pooled samples, we applied the pipeline to DNA samples from single individuals. We used the frequency output to infer whether a given insertion was present in the genome considered. Insertions were considered identical if their estimated location was within 200 bp of each other [44]. Any insertion with an estimated frequency higher than 0.8 was treated as a homozygous; insertions with an estimated frequency of < 0.2 were considered errors and insertions with an intermediate frequency were called as heterozygous. To test PoPoolationTE’s ability to correctly distinguish heterozygote and homozygous insertions we ran the pipeline on two *C. rubella* accessions (*cr1gr1* and *JGI*) that have been selfed for multiple generations in the greenhouse, as well as on a hybrid sample created by merging the sequences of both samples. If the programme can correctly infer homozygous and heterozygous insertions, we expect almost all insertions in the pure samples to be fixed and so have an inferred frequency of 1 and the hybrid to show an increase of calls around 0.5. Indeed, this is what we observe (Additional file 4: Figure S3). In the two highly selfed samples 88% and 82% of all insertions had an inferred frequency of 1, which was reduced in the hybrid to 61%. The shape of the count distribution also provides justification for using 0.8 as a cut-off. In the two highly selfed samples, the counts remain very low until around 0.8 where there is an increase (although this is less clear in the *JGI* sample). Moreover, to further assess whether our conclusions were robust to our homo- heterozygous individual-based calling approach we redid the copy number comparison across species using the raw frequency estimates from all individuals and we find that patterns do not change (Kruskal-Wallis chi-squared = 29.6582, df = 2, p < 0.00001). Finally, to test how our individual-based approach compared with the pooled approach, we constructed pooled sequence samples by merging the sequences from all individuals of a species into one pooled sample. Again, we find that are conclusions about TE abundance in the three species do not change (Kruskal-Wallis chi-squared = 24.0303, df = 2, p < 0.00001).

Venn diagrams of unique and shared insertions were generated using VENNY [57]. Using the gene annotation from the *C. rubella* reference genome [39], we calculated the distance to the closest gene for all insertions. Finally, we calculated the number of insertions in 100 bp bins.

### Frequency distributions

We used the presence or absence of all identified insertions to calculate frequency distributions for all three species. For the self-compatible species *C. rubella* and *C. orientalis* all insertions were assumed to be homozygous. As mentioned in the Introduction, the effective selfing rate in *C. rubella* is around 0.90–0.97 [42] and whereas selfing rate in *C. orientalis* has not been quantified but very low allozyme variability suggests that the species is predominately selfing [38]. In this case, the frequency for each insertion is the number of sampled individuals in which the insertion was detected, divided by the total number individuals. For outcrossing *C. grandiflora*, we treated each haplotype as an independent sample. Frequency calculations were restricted to 8 randomly chosen individuals for each selfing species, and half of that (4) for *C. grandiflora*.

## Availability of supporting data

The datasets supporting the results of this article are available for download. *De novo* assemblies are available on CoGe (https://genomevolution.org/CoGe/) for *C. orientalis* (Genome ID 24033), *C. grandiflora* (Genome ID 24068), and *N. paniculata* (Genome ID 24067). Paired-end sequences are available on the Sequence Read Archive (http://www.ncbi.nlm.nih.gov/sra) for *C. orientalis* (Accession number SRP041585), *C. grandiflora* (Accession number SRP044121), and *C. rubella* (Accession number PRJEB6689).

## Electronic supplementary material

Additional file 1: Figure S1: Total TE copy number in sampled individuals in the three *Capsella* species. (PNG 63 KB)

Additional file 2: Figure S2: Average TE copy number in the three *Capsella* species. Each species was mapped to its own genome (a) and to the *Neslia paniculata* assembly (b) using a TE database based on *Arabidopsis thaliana* and *Arabidopsis lyrata*. The difference between *C. rubella* and the other species in (a) is exaggerated by the higher quality of the *C. rubella* reference genome compared with the Illumina-only *de novo* assemblies of the other species. Error bars are ± 1 standard error. (PNG 114 KB)

Additional file 3: Table S1: Origins of sequenced *Capsella* samples. **Table S2**. Assembly statistics for *C. orientalis de novo* assembly. (DOC 63 KB)

Additional file 4: Figure S3: Counts of inferred TE frequency for two highly selfed accessions (cr1gr1 and JGI), as well as on a hybrid sample created by merging the sequences of both samples. The Y-axis is cut at 300 to highlight the increase in the number of insertions of intermediate frequencies inferred in the hybrid sample. (PNG 163 KB)
