# Supplementary figures and images for: Mating system shifts and transposable element evolution in the plant genus Capsella
Source: BMC Genomics. 2014 Jul 16;15(1):602. doi: 10.1186/1471-2164-15-602 (PMC4112209; doi:10.1186/1471-2164-15-602)

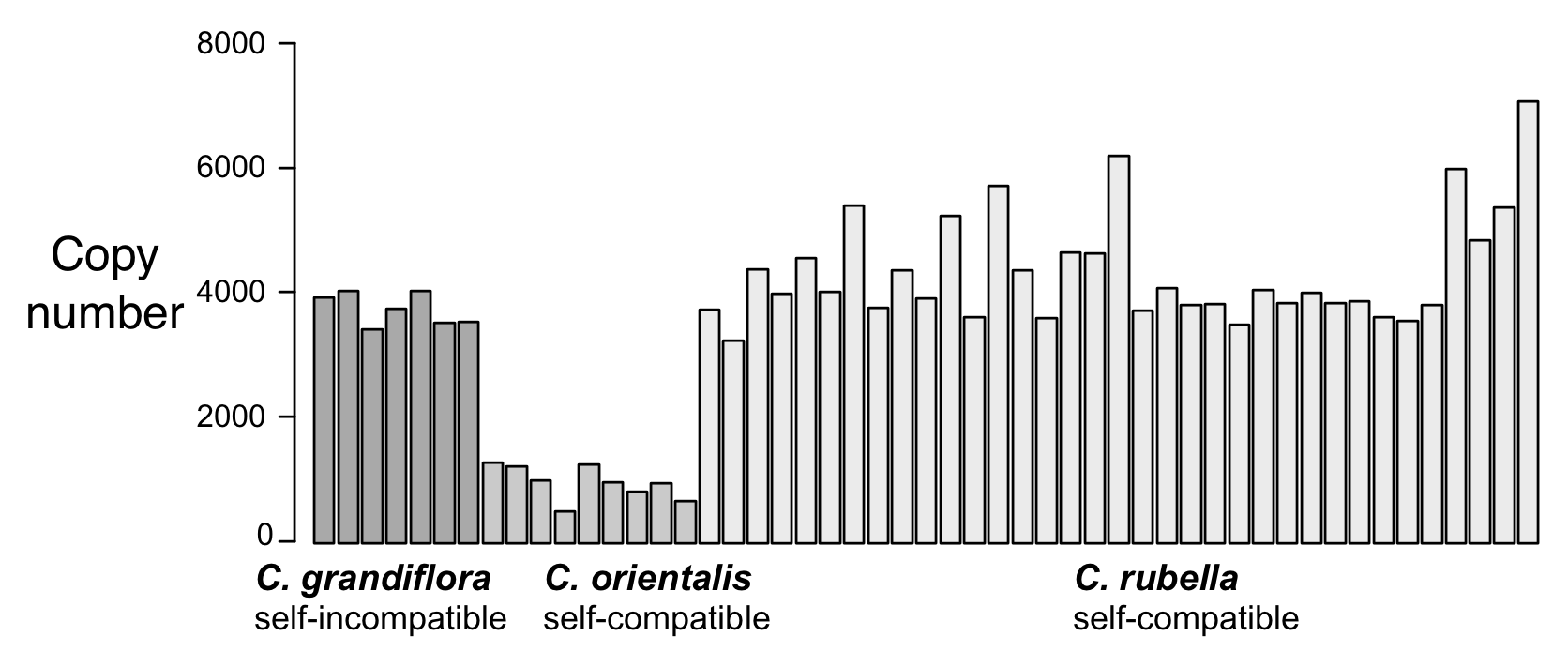

Supplement: Supplementary file 1 — Additional file 1: Figure S1: Total TE copy number in sampled individuals in the three Capsella species. (PNG 63 KB) [file 12864_2013_6287_MOESM1_ESM.png]

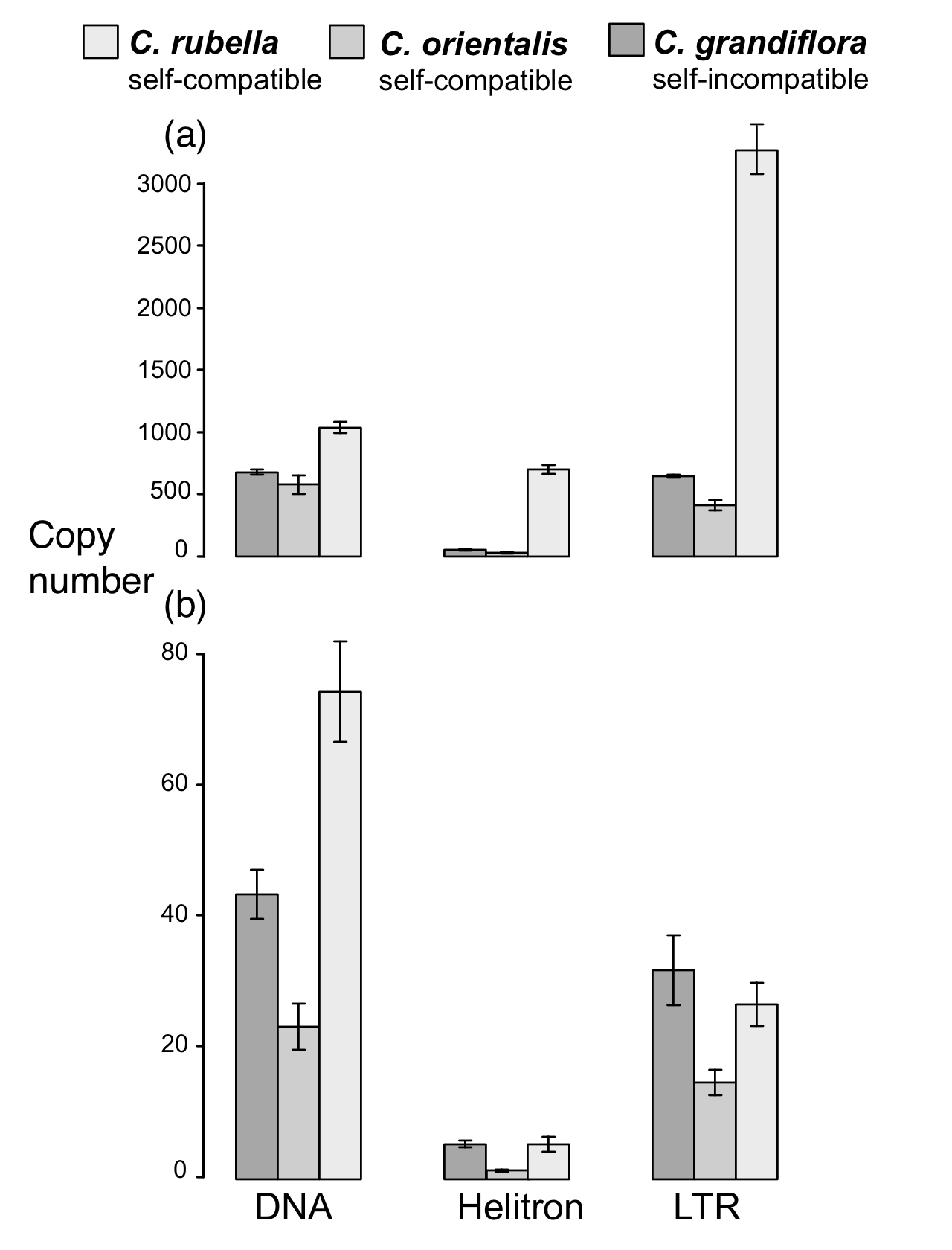

Supplement: Supplementary file 2 — Additional file 2: Figure S2: Average TE copy number in the three Capsella species. Each species was mapped to its own genome (a) and to the Neslia paniculata assembly (b) using a TE database based on Arabidopsis thaliana and Arabidopsis lyrata. The difference between C. rubella and the other species in (a) is exaggerated by the higher quality of the C. rubella reference genome compared with the Illumina-only de novo assemblies of the other species. Error bars are ± 1 standard error. (PNG 114 KB) [file 12864_2013_6287_MOESM2_ESM.png]

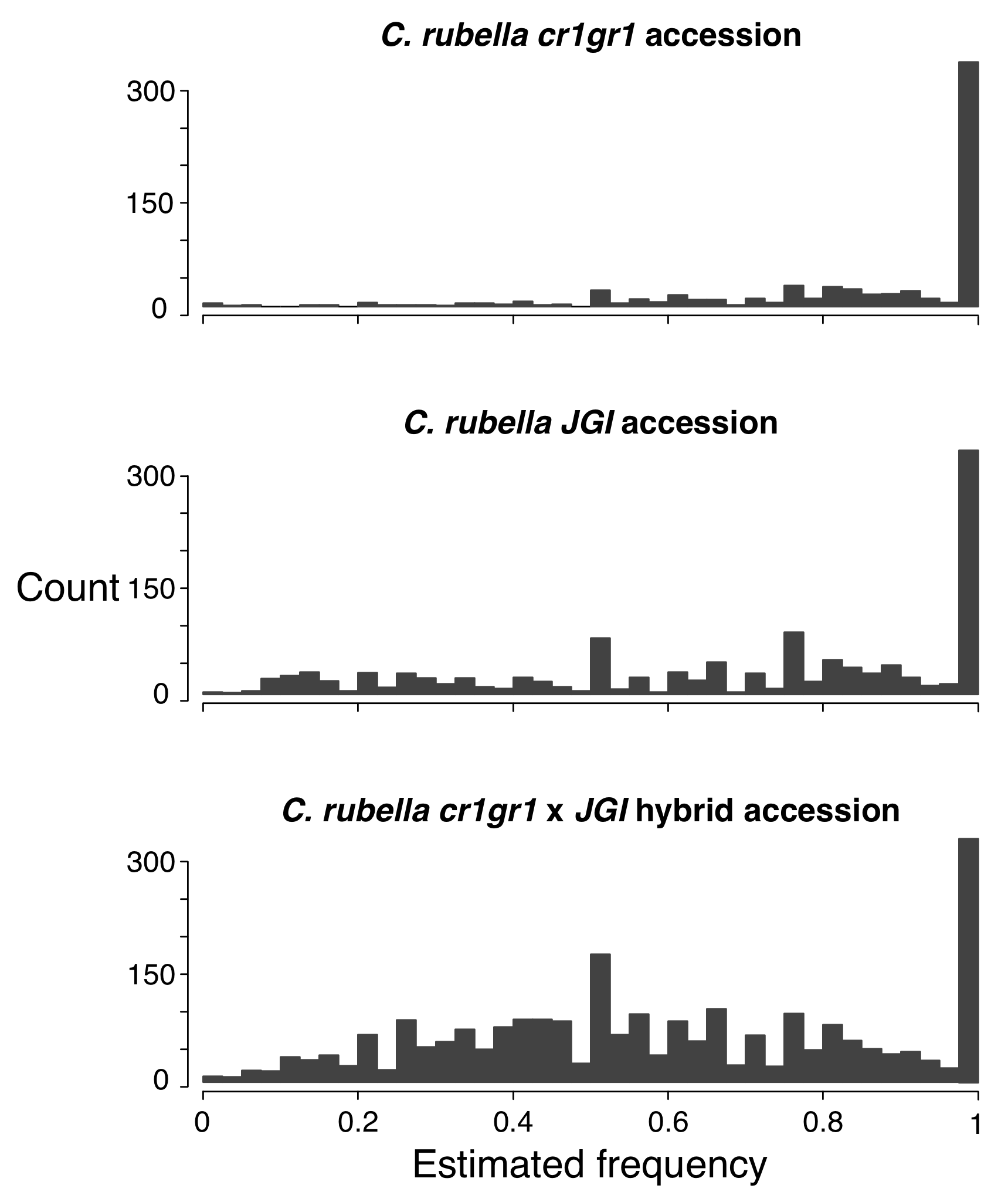

Supplement: Supplementary file 4 — Additional file 4: Figure S3: Counts of inferred TE frequency for two highly selfed accessions (cr1gr1 and JGI), as well as on a hybrid sample created by merging the sequences of both samples. The Y-axis is cut at 300 to highlight the increase in the number of insertions of intermediate frequencies inferred in the hybrid sample. (PNG 163 KB) [file 12864_2013_6287_MOESM4_ESM.png]
