# Supplementary material for: Mating system shifts and transposable element evolution in the plant genus Capsella
Source: BMC Genomics. 2014 Jul 16;15(1):602. doi: 10.1186/1471-2164-15-602 (PMC4112209; doi:10.1186/1471-2164-15-602)
Supplement: Supplementary file 3 — Additional file 3: Table S1: Origins of sequenced Capsella samples. Table S2. Assembly statistics for C. orientalis de novo assembly. (DOC 63 KB) [file 12864_2013_6287_MOESM3_ESM.doc]

**Additional file 3**

**Table S1 Origins of sequenced *Capsella*** samples

| **Species** | **Accession** | **Latitude (ºN)** | **Longitude (ºE)** | **Notes** |
| --- | --- | --- | --- | --- |
| *C. grandiflora* | 83.17 | 38.4379 | 21.42431667 |  |
| *C. grandiflora* | 85.33 | 39.55521667 | 20.91641667 |  |
| *C. grandiflora* | AxE | n/a | n/a | Cross betweeen individuals918/8 and Cg2e, two Corfu populations |
| *C. grandiflora* | 103.17 | 39.51838333 | 21.56091667 |  |
| *C. grandiflora* | 5a | 39.705025 | 19.7573444 |  |
| *C. grandiflora* | 91.2 | 39.86715 | 20.70708333 |  |
| *C. grandiflora* | 95.15 | 39.14541667 | 20.05816667 |  |
| *C. grandiflora* | 86.8 | 39.01723333 | 20.13193333 |  |
| *C. rubella* | 690 | 36.15 | -5.58 |  |
| *C. rubella* | 697 | 44.1336 | 10.2144 |  |
| *C. rubella* | 698 | 44.1336 | 10.2144 |  |
| *C. rubella* | 762 | 37.966667 | 23.716667 |  |
| *C. rubella* | 844 | 35.202554 | 24.233091 |  |
| *C. rubella* | 879 | 35.29 | 24.42 |  |
| *C. rubella* | 907 | 39.666667 | 19.8 |  |
| *C. rubella* | 925 | 39.666667 | 20.85 |  |
| *C. rubella* | 984 | 39.5 | 3 |  |
| *C. rubella* | 1207 | 28.316667 | -16.566667 |  |
| *C. rubella* | 1208 | 28.316667 | -16.566667 |  |
| *C. rubella* | 1209 | 28.316667 | -16.566667 |  |
| *C. rubella* | 1215 | 28.316667 | -16.566667 |  |
| *C. rubella* | 1311 | 42.8833333 | -0.1 |  |
| *C. rubella* | 1377 | -34.6666667 | -58.5 |  |
| *C. rubella* | 1453 | 43.466667 | 11.033333 |  |
| *C. rubella* | 774 | 41.8333333 | 16 |  |
| *C. rubella* | 86IT1 | 40.62 | 14.37 |  |
| *C. rubella* | 1GR1 | 37.742485 | 26.81958 |  |
| *C. rubella* | 1574-1 | 41.6 | 8.983333 |  |
| *C. rubella* | 1407-8 | 35.183333 | 24.233333 |  |
| *C. rubella* | 1504-11 | 28.666667 | -17.866667 |  |
| *C. rubella* | 1575-1 | 41.383333 | 9.166667 |  |
| *C. rubella* | 1249-11 | 38.7833333 | -9.3833333 |  |
| *C. orientalis* | 1719-3 | 48.35 | 88.26 |  |
| *C. orientalis* | 1719-4 | 48.35 | 88.26 |  |
| *C. orientalis* | 1979-1 | 51.07 | 81.48 |  |
| *C. orientalis* | 1979-7 | 51.07 | 81.48 |  |
| *C. orientalis* | 1981-10 | 51.08 | 81.36 |  |
| *C. orientalis* | 1981-6 | 51.08 | 81.36 |  |
| *C. orientalis* | 1985-1 | 53.21 | 83.44 |  |
| *C. orientalis* | 1985-5 | 53.21 | 83.44 |  |
| *C. orientalis* | 2008-1 | 46.37 | 90.52 |  |
| *C. orientalis* | 2008-2 | 46.37 | 90.52 |  |

**Table S2 Assembly statistics for *C. orientalis de novo*** assembly

| Number of contigs | 23,659 |
| --- | --- |
| Total number of base pairs (bp) | 143,780,143 bp |
| Shortest contig | 500 bp |
| Longest contig | 647,746 bp |
| Average GC% | 35.0 |
| Non-ACGT bases | 2,655,457 bp |
| N50 | 25,186 bp |
